# Supplementary material for: Pyruvate Dehydrogenase Kinase 1 inhibition mediated oxidative phosphorylation enhancement in cartilage promotes osteoarthritis progression
Source: BMC Musculoskelet Disord. 2023 Jul 20;24:597. doi: 10.1186/s12891-023-06585-6 (PMC10357736; doi:10.1186/s12891-023-06585-6)
Supplement: Supplementary file 1 — Additional file 1. [file 12891_2023_6585_MOESM1_ESM.zip › BP.Gsea.1653623667859/GOBP_ENDOPLASMIC_RETICULUM_ORGANIZATION.html]

Details for gene set GOBP\_ENDOPLASMIC\_RETICULUM\_ORGANIZATION[GSEA]

|  || Dataset | 55457\_collapsed\_to\_symbols.cls.cls #OA\_versus\_normal.cls.cls #OA\_versus\_normal\_repos |
| Phenotype | cls.cls#OA\_versus\_normal\_repos |
| Upregulated in class | OA |
| GeneSet | GOBP\_ENDOPLASMIC\_RETICULUM\_ORGANIZATION |
| Enrichment Score (ES) | 0.36047992 |
| Normalized Enrichment Score (NES) | 1.5685457 |
| Nominal p-value | 0.0078125 |
| FDR q-value | 0.28941688 |
| FWER p-Value | 1.0 |
Table: GSEA Results Summary

  

Fig 1: Enrichment plot: GOBP\_ENDOPLASMIC\_RETICULUM\_ORGANIZATION      
 Profile of the Running ES Score & Positions of GeneSet Members on the Rank Ordered List

  

| SYMBOL | TITLE | RANK IN GENE LIST | RANK METRIC SCORE | RUNNING ES | CORE ENRICHMENT || 1 | GET1 | guided entry of tail-anchored proteins factor 1 [Source:HGNC Symbol;Acc:HGNC:12790] | 159 | 0.852 | 0.0239 | Yes |
| 2 | MOSPD3 | motile sperm domain containing 3 [Source:HGNC Symbol;Acc:HGNC:25078] | 188 | 0.829 | 0.0570 | Yes |
| 3 | WDR83OS | WD repeat domain 83 opposite strand [Source:HGNC Symbol;Acc:HGNC:30203] | 239 | 0.791 | 0.0867 | Yes |
| 4 | EMC9 | ER membrane protein complex subunit 9 [Source:HGNC Symbol;Acc:HGNC:20273] | 276 | 0.768 | 0.1166 | Yes |
| 5 | REEP5 | receptor accessory protein 5 [Source:HGNC Symbol;Acc:HGNC:30077] | 356 | 0.722 | 0.1413 | Yes |
| 6 | TLE6 | "TLE family member 6, subcortical maternal complex member [Source:HGNC Symbol;Acc:HGNC:30788]" | 385 | 0.711 | 0.1694 | Yes |
| 7 | UBL4A | ubiquitin like 4A [Source:HGNC Symbol;Acc:HGNC:12505] | 464 | 0.675 | 0.1920 | Yes |
| 8 | EMC7 | ER membrane protein complex subunit 7 [Source:HGNC Symbol;Acc:HGNC:24301] | 468 | 0.673 | 0.2204 | Yes |
| 9 | LMAN2 | "lectin, mannose binding 2 [Source:HGNC Symbol;Acc:HGNC:16986]" | 485 | 0.666 | 0.2475 | Yes |
| 10 | LMAN2L | "lectin, mannose binding 2 like [Source:HGNC Symbol;Acc:HGNC:19263]" | 507 | 0.656 | 0.2738 | Yes |
| 11 | TRAM1 | translocation associated membrane protein 1 [Source:HGNC Symbol;Acc:HGNC:20568] | 569 | 0.633 | 0.2960 | Yes |
| 12 | BNIP1 | BCL2 interacting protein 1 [Source:HGNC Symbol;Acc:HGNC:1082] | 824 | 0.563 | 0.3002 | Yes |
| 13 | REEP4 | receptor accessory protein 4 [Source:HGNC Symbol;Acc:HGNC:26176] | 901 | 0.542 | 0.3174 | Yes |
| 14 | EMC1 | ER membrane protein complex subunit 1 [Source:HGNC Symbol;Acc:HGNC:28957] | 1067 | 0.502 | 0.3259 | Yes |
| 15 | EMC2 | ER membrane protein complex subunit 2 [Source:HGNC Symbol;Acc:HGNC:28963] | 1096 | 0.497 | 0.3448 | Yes |
| 16 | GET3 | "guided entry of tail-anchored proteins factor 3, ATPase [Source:HGNC Symbol;Acc:HGNC:752]" | 1161 | 0.485 | 0.3605 | Yes |
| 17 | EMC3 | ER membrane protein complex subunit 3 [Source:HGNC Symbol;Acc:HGNC:23999] | 1624 | 0.410 | 0.3420 | No |
| 18 | RAB3GAP1 | RAB3 GTPase activating protein catalytic subunit 1 [Source:HGNC Symbol;Acc:HGNC:17063] | 1766 | 0.389 | 0.3475 | No |
| 19 | ESYT1 | extended synaptotagmin 1 [Source:HGNC Symbol;Acc:HGNC:29534] | 2129 | 0.347 | 0.3341 | No |
| 20 | TOR1B | torsin family 1 member B [Source:HGNC Symbol;Acc:HGNC:11995] | 2355 | 0.320 | 0.3302 | No |
| 21 | DOP1B | DOP1 leucine zipper like protein B [Source:HGNC Symbol;Acc:HGNC:1291] | 2687 | 0.280 | 0.3163 | No |
| 22 | TRDN | triadin [Source:HGNC Symbol;Acc:HGNC:12261] | 2731 | 0.277 | 0.3248 | No |
| 23 | EMC6 | ER membrane protein complex subunit 6 [Source:HGNC Symbol;Acc:HGNC:28430] | 2839 | 0.266 | 0.3278 | No |
| 24 | CASQ1 | calsequestrin 1 [Source:HGNC Symbol;Acc:HGNC:1512] | 3141 | 0.236 | 0.3144 | No |
| 25 | MIA3 | MIA SH3 domain ER export factor 3 [Source:HGNC Symbol;Acc:HGNC:24008] | 3550 | 0.198 | 0.2910 | No |
| 26 | REEP1 | receptor accessory protein 1 [Source:HGNC Symbol;Acc:HGNC:25786] | 3589 | 0.195 | 0.2963 | No |
| 27 | CAMLG | calcium modulating ligand [Source:HGNC Symbol;Acc:HGNC:1471] | 3668 | 0.188 | 0.2983 | No |
| 28 | REEP2 | receptor accessory protein 2 [Source:HGNC Symbol;Acc:HGNC:17975] | 3899 | 0.165 | 0.2874 | No |
| 29 | VAPB | VAMP associated protein B and C [Source:HGNC Symbol;Acc:HGNC:12649] | 3905 | 0.164 | 0.2940 | No |
| 30 | POLR2M | RNA polymerase II subunit M [Source:HGNC Symbol;Acc:HGNC:14862] | 3907 | 0.164 | 0.3009 | No |
| 31 | ATL2 | atlastin GTPase 2 [Source:HGNC Symbol;Acc:HGNC:24047] | 4025 | 0.154 | 0.2983 | No |
| 32 | RTN3 | reticulon 3 [Source:HGNC Symbol;Acc:HGNC:10469] | 4508 | 0.112 | 0.2655 | No |
| 33 | TRAM2 | translocation associated membrane protein 2 [Source:HGNC Symbol;Acc:HGNC:16855] | 4913 | 0.079 | 0.2374 | No |
| 34 | CAV2 | caveolin 2 [Source:HGNC Symbol;Acc:HGNC:1528] | 4924 | 0.078 | 0.2400 | No |
| 35 | STX18 | syntaxin 18 [Source:HGNC Symbol;Acc:HGNC:15942] | 4968 | 0.075 | 0.2398 | No |
| 36 | BAG6 | BAG cochaperone 6 [Source:HGNC Symbol;Acc:HGNC:13919] | 5023 | 0.071 | 0.2386 | No |
| 37 | SEC61A1 | SEC61 translocon subunit alpha 1 [Source:HGNC Symbol;Acc:HGNC:18276] | 5378 | 0.041 | 0.2128 | No |
| 38 | TMED2 | transmembrane p24 trafficking protein 2 [Source:HGNC Symbol;Acc:HGNC:16996] | 5709 | 0.015 | 0.1877 | No |
| 39 | SEC31A | "SEC31 homolog A, COPII coat complex component [Source:HGNC Symbol;Acc:HGNC:17052]" | 6021 | -0.006 | 0.1637 | No |
| 40 | ARL6IP1 | ADP ribosylation factor like GTPase 6 interacting protein 1 [Source:HGNC Symbol;Acc:HGNC:697] | 6025 | -0.006 | 0.1638 | No |
| 41 | TMEM33 | transmembrane protein 33 [Source:HGNC Symbol;Acc:HGNC:25541] | 6085 | -0.011 | 0.1597 | No |
| 42 | EMC8 | ER membrane protein complex subunit 8 [Source:HGNC Symbol;Acc:HGNC:7864] | 6104 | -0.013 | 0.1588 | No |
| 43 | LMAN1 | "lectin, mannose binding 1 [Source:HGNC Symbol;Acc:HGNC:6631]" | 6361 | -0.032 | 0.1402 | No |
| 44 | VCPIP1 | valosin containing protein interacting protein 1 [Source:HGNC Symbol;Acc:HGNC:30897] | 7709 | -0.135 | 0.0410 | No |
| 45 | RAB3GAP2 | RAB3 GTPase activating non-catalytic protein subunit 2 [Source:HGNC Symbol;Acc:HGNC:17168] | 7957 | -0.152 | 0.0282 | No |
| 46 | TMEM38B | transmembrane protein 38B [Source:HGNC Symbol;Acc:HGNC:25535] | 8246 | -0.175 | 0.0132 | No |
| 47 | DNM1L | dynamin 1 like [Source:HGNC Symbol;Acc:HGNC:2973] | 8499 | -0.194 | 0.0019 | No |
| 48 | VAPA | VAMP associated protein A [Source:HGNC Symbol;Acc:HGNC:12648] | 8854 | -0.221 | -0.0163 | No |
| 49 | SHTN1 | shootin 1 [Source:HGNC Symbol;Acc:HGNC:29319] | 8966 | -0.229 | -0.0152 | No |
| 50 | TOR1AIP2 | torsin 1A interacting protein 2 [Source:HGNC Symbol;Acc:HGNC:24055] | 9133 | -0.242 | -0.0178 | No |
| 51 | SEC16A | "SEC16 homolog A, endoplasmic reticulum export factor [Source:HGNC Symbol;Acc:HGNC:29006]" | 9434 | -0.268 | -0.0298 | No |
| 52 | LPCAT3 | lysophosphatidylcholine acyltransferase 3 [Source:HGNC Symbol;Acc:HGNC:30244] | 9461 | -0.270 | -0.0203 | No |
| 53 | STEEP1 | STING1 ER exit protein 1 [Source:HGNC Symbol;Acc:HGNC:26239] | 9592 | -0.282 | -0.0184 | No |
| 54 | EIF2AK3 | eukaryotic translation initiation factor 2 alpha kinase 3 [Source:HGNC Symbol;Acc:HGNC:3255] | 10025 | -0.324 | -0.0383 | No |
| 55 | GAK | cyclin G associated kinase [Source:HGNC Symbol;Acc:HGNC:4113] | 10301 | -0.350 | -0.0448 | No |
| 56 | PEX5 | peroxisomal biogenesis factor 5 [Source:HGNC Symbol;Acc:HGNC:9719] | 10737 | -0.398 | -0.0617 | No |
| 57 | SGTA | small glutamine rich tetratricopeptide repeat co-chaperone alpha [Source:HGNC Symbol;Acc:HGNC:10819] | 11185 | -0.455 | -0.0772 | No |
| 58 | TMCC1 | transmembrane and coiled-coil domain family 1 [Source:HGNC Symbol;Acc:HGNC:29116] | 11308 | -0.472 | -0.0666 | No |
| 59 | LMAN1L | "lectin, mannose binding 1 like [Source:HGNC Symbol;Acc:HGNC:6632]" | 11430 | -0.492 | -0.0551 | No |
| 60 | EMC10 | ER membrane protein complex subunit 10 [Source:HGNC Symbol;Acc:HGNC:27609] | 11497 | -0.501 | -0.0389 | No |
| 61 | SEC31B | "SEC31 homolog B, COPII coat complex component [Source:HGNC Symbol;Acc:HGNC:23197]" | 11690 | -0.531 | -0.0312 | No |
| 62 | RTN4 | reticulon 4 [Source:HGNC Symbol;Acc:HGNC:14085] | 12017 | -0.596 | -0.0313 | No |
| 63 | CERT1 | ceramide transporter 1 [Source:HGNC Symbol;Acc:HGNC:2205] | 12463 | -0.732 | -0.0348 | No |
| 64 | CCDC47 | coiled-coil domain containing 47 [Source:HGNC Symbol;Acc:HGNC:24856] | 12495 | -0.746 | -0.0054 | No |
| 65 | SMPD4 | sphingomyelin phosphodiesterase 4 [Source:HGNC Symbol;Acc:HGNC:32949] | 12702 | -0.866 | 0.0154 | No |
Table: GSEA details [plain text format]

  

Fig 2: GOBP\_ENDOPLASMIC\_RETICULUM\_ORGANIZATION      
 Blue-Pink O' Gram in the Space of the Analyzed GeneSet

  

Fig 3: GOBP\_ENDOPLASMIC\_RETICULUM\_ORGANIZATION: Random ES distribution      
 Gene set null distribution of ES for **GOBP\_ENDOPLASMIC\_RETICULUM\_ORGANIZATION**
